# Mycobacterioses Induced by *Mycobacterium abscessus*: Case Studies Indicating the Importance of Molecular Analysis for the Identification of Antibiotic Resistance

**DOI:** 10.3390/antibiotics11070873

**Published:** 2022-06-28

**Authors:** Lenka Ryskova, Radka Bolehovska, Rudolf Kukla, Michal Svarc, Alzbeta Zavrelova, Hubert Vanicek, Ivo Pavlik, Pavel Bostik

**Affiliations:** 1 Institute of Clinical Microbiology, Faculty of Medicine in Hradec Kralove, Charles University, 50003 Hradec Kralove, Czech Republic; lenka.ryskova@fnhk.cz (L.R.); radka.bolehovska@fnhk.cz (R.B.); rudolf.kukla@fnhk.cz (R.K.); 2 University Hospital, Charles University, 50005 Hradec Kralove, Czech Republic; michal.svarc@fnhk.cz (M.S.); alzbeta.zavrelova@fnhk.cz (A.Z.); hubert.vanicek@fnhk.cz (H.V.); 3 Department of Pneumology, Faculty of Medicine in Hradec Kralove, Charles University, 50005 Hradec Kralove, Czech Republic; 4 4th Department of Internal Medicine, Faculty of Medicine in Hradec Kralove, Charles University, 50005 Hradec Kralove, Czech Republic; 5 Department of Pediatrics, Faculty of Medicine in Hradec Kralove, Charles University, 50005 Hradec Kralove, Czech Republic; 6 Faculty of Regional Development and International Studies, Mendel University in Brno, tr. Generala Piky 7, 61300 Brno, Czech Republic; ivo.pavlik@mendelu.cz

**Keywords:** nontuberculous mycobacteria, rapidly growing mycobacteria, macrolide resistance, *erm*(41) gene, multiresistance

## Abstract

Mycobacterioses are less frequently occurring but serious diseases. In recent years, at a global level, the incidence of mycobacterioses induced by the rapidly growing species *Mycobacterium abscessus* (*M. a.*), which is considered to be the most resistant to antibiotics and most difficult to treat, has been on the rise. Correct identification to the level of the subspecies (*M. a. abscessus*, *M. a. massiliense*, and *M. a. bolletii*) and determination of its sensitivity to macrolides, which are the basis of combination therapy, are of principal importance for the management of the disease. We describe five cases of mycobacterioses caused by *M. a.*, where the sequencing of select genes was performed to identify the individual subspecies and antibiotic resistance. The analysis of the *rpo*B gene showed two isolates each of *M. a. abscessus* and *M. a. massiliense* and one isolate of *M. a. bolletii*. The complete (full length) *erm*(41) gene responsible for the development of inducible resistance to macrolides was demonstrated in both *M. a. abscessus* and *M. a. bolletii* isolates. A partially deleted and non-functional *erm*(41) gene was demonstrated in *M. a. massiliense* isolates. The subsequent sequencing of the full length *erm*(41) gene products showed, however, the mutation (T28→C) in both isolates of *M. a. abscessus*, causing a loss of the function and preserved sensitivity to macrolides. The antibiotic sensitivity testing confirmed that both the isolates of *M. a. abscessus* and *M. a. massiliense* were sensitive to clarithromycin even after prolonged 14-day incubation. The inducible resistance to clarithromycin was maintained only in *M. a. bolletii*. Thus, the sequence analysis of the *erm*(41) gene can reliably identify the preservation of sensitivity to macrolides and serve as an important tool in the establishment of therapeutic regimens in cases of infections with *M. abscessus*.

## 1. Introduction

*Mycobacterium abscessus* (*M. a.*) is a rapidly growing nontuberculous *Mycobacterium* (NTM) belonging to a species of serious clinical importance. Since the beginning of the new millennium, an increase in the occurrence of mycobacterioses has been observed globally [1,2], which is primarily due to pulmonary infections (80–90%), and, to a lesser extent, infections of the lymph nodes, soft tissues, bones, joints, as well as disseminated diseases and catheter-related infections [3]. The increase in the number of NTM diseases is generally accredited to changes in population structure due to ageing, which leads to an increase in numbers of people with comorbidities including immunosuppression. Climate change also leads to an increase in numbers of sources of NTM in the environment and, interestingly, changes of hygiene habits among population may increase the exposure to NTM. Thus, showering is increasingly becoming more preferred to bathing, while shower heads are often colonized by NTM, specifically by *M. avium* [4,5]. Finally, yet importantly, the increased incidence of NTM diagnosis is due to both improvements in laboratory diagnostics and an increase in the general knowledge concerning NTM among physicians [6].

A higher prevalence of mycobacterioses has been recorded mainly in North America, Asia, and Australia, ranging from 7.9 to 14.1/100,000 inhabitants [1]. In Europe, a lower prevalence of newly diagnosed cases has been observed, ranging between 0.2 and 2.9/100,000 inhabitants [2]. Around the world, the *M. avium* complex (MAC) has remained the most common causative agent of mycobacterioses, while the frequency of other NTM species has shown geographic variability [7]. In Europe, the most frequently isolated NTM species after MAC are *M. kansasii*, *M. xenopi*, and *M. malmoense*. This is in contrast to the epidemiological data from North America, Asia, and Australia, where *M. a.* is the second most frequent causative agent of mycobacterioses [7,8].

In the Czech Republic, according to the Information System of Bacillary Tuberculosis (ISBT), the situation is similar to other European countries, where MAC members are the most common causative agents of mycobacterioses, followed by *M. kansasii* and *M. xenopi*. Infections caused by *M. a.* are, however, recorded only scarcely. Thus, during the years 2012–2018, *M. a.* was diagnosed according to American Thoracic Society (ATS) Criteria in only four (1.32%) patients with mycobacterioses [9]. Nevertheless, in light of the global rise of the mycobacterioses induced by *M. a.* [10], especially in the population of the patients with cystic fibrosis (CF), this trend may be likely expected in the Czech Republic as well.

Treatment of mycobacterioses is generally complicated as it requires the administration of a combination of antibiotics for a period of at least 12 months owing to the conversion of the sputum to cultivation negativity, according to the recommendation of the ATS [3,11]. However, treatment of diseases caused by *M. a.* has been shown to be even more difficult, as this species is increasingly resistant to antibiotics. *M. a.* is intrinsically resistant not only to the classical anti-tuberculous drugs, but also to most of the currently available antibiotics. This resistance is attributed predominantly to the impermeable cell envelope, enzymatic deactivation of drugs, and drug efflux. Susceptibility testing should be performed against a number of antibiotics, including clarithromycin, amikacin, tigecycline, imipenem, cefoxitin, clofazimine, doxycycline, ciprofloxacin, moxifloxacin. The final antibiotic selection should be then guided by drug susceptibility testing. Macrolides represent the critical antibiotic in the combinatory therapy of *M. abscessus* infections, and the resistance against them may lead to therapy failure [12]. In the treatment of mycobacterioses induced by *M. a.*, an introductory combination of at least three effective antibiotics is recommended in the strains sensitive to macrolides, and a combination of at least four antibiotics in resistant strains for a period of at least 1–3 months. This intensive phase usually includes oral macrolide, intravenous amikacin, and one or more of the following: intravenous tigecycline, imipenem, or cefoxitin. This should be followed by a long-term therapeutic regimen with 2–3 effective substances for a period of at least 12 months [13]. The continuation phase can include oral macrolide, inhaled amikacin, moxifloxacin, linezolid, and clofazimine. Nevertheless, the recommended procedures are based on very limited experience, and the optimal approach is still not known [14].

At present three subspecies of M. a. are known: *M. a*. ssp. *abscessus* (*M. a. abscessus*), *M. a*. ssp. *bolletii* (*M. a. bolletii*), and *M. a*. ssp. *massiliense* (*M. a. massiliense*). Infections caused by *M. a. abscessus* and *M. a. bolletii* are generally associated with a clinically worse course and a frequent failure of therapy mainly due to their common resistance to macrolides. This resistance can be constitutive or inducible [15]. Constitutive resistance in *M. a*. occurs rarely and develops on the basis of spontaneous point mutation in the region of the *rrl* gene with subsequent selection of the strain during therapy with macrolides. Inducible resistance is connected with the presence of the functional *erm*(41) gene, which codes for the methylase that alters the target point for the macrolides on the ribosome [16,17]. The inducible resistance typically does not manifest by culture method during the standard incubation period in the in vitro tests for the determination of sensitivity to macrolides, but is observed later on after an extension of the incubation period to 14 days [18,19]. The functional gene for inducible resistance to macrolides is carried by most *M. a. abscessus* and *M. a. bolletii* isolates. *M. a. massiliense*, however, carry a deletion in the *erm*(41), and their sensitivity to macrolides is preserved, leading to better treatment outcomes [20]. At the same time, it has been found that a number of *M. a. abscessus* isolates carry mutations in the *erm*(41) gene, leading to its loss of function and preserved sensitivity to macrolides [12].

Thus, to apply an effective therapeutic regimen in such infections, it is essential to identify the causative agent to the level of the subspecies of *M. a.* and further determine its sensitivity to macrolides.

This study describes five cases of otherwise rare clinically important detections of *M. a.* in patients treated at the University Hospital in Hradec Kralove (Czech Republic), their disease and treatment history, and shows the importance of molecular analysis for the subspecies identification and determination of antibiotic sensitivity.

## 2. Results

### 2.1. Case Studies

#### 2.1.1. Case 1

A 19-year-old man with CF; the first detection of *M. a.* in the sputum was recorded during a periodical monitoring of the principal disease in August 2013. The clinical condition of the patient was evaluated as stable, without progression, and without a need for additional therapy. Nevertheless, subsequent control examinations of the sputum repeatedly demonstrated *M. a*. The isolate was further determined as *M. a. bolletii* (Str 1). In May 2014, the clinical function of the lungs deteriorated with a corresponding finding on high resolution computed tomography (HRCT). After excluding other causes of infection and the demonstration of *M. a. bolletii* in bronchoalveolar lavage fluid (BAL), an introductory 6-week intravenous therapy was initiated using a combination of amikacin, imipenem, and clarithromycin, which was followed by an oral continuing therapy with doxycycline, clarithromycin, and ciprofloxacin (selection and dosage of antibiotics according to Griffith et al., 2007 [3]). After 2 months of oral therapy, progression of the pathological findings in the lungs was observed on CT, with further worsening of clinical symptoms. The cultivation for *M. a. bolletii* was positive again in both the sputum and BAL. The Minimum Inhibitory Concentration (MIC) examination revealed that the Str 1, which was originally sensitive to macrolides, was now resistant. Intravenous therapy was initiated again with the combination of imipenem, tigecycline, amikacin, and azithromycin (for immunomodulatory effect) for a period of 3 months. After that, the patient was treated with oral azithromycin and doxycycline, complemented with inhaled amikacin. Partial regression of the largest infiltrates was observed, and the clinical condition of the patient improved. However, the culture positivity for *M. a*. *bolletii* persisted during the entire year of 2015. According to the recommendation of the European Cystic Fibrosis Society (ECFS), clofazimine was added in December 2015. Conversion of the sputum to *M. a. bolletii* cultivation negativity occurred in May 2017, which was partially due to an improvement of the patient’s compliance. Therapy was terminated in the year 2018.

#### 2.1.2. Case 2

A 33-year-old patient was diagnosed with myelomonocytic leukaemia in October 2013. After allogeneic transplantation in April 2014, serious Graft versus Host Disease (GvHD) of the lungs developed and was treated with corticoids, methotrexate, and rituximab. In August 2016, pulmonary aspergillosis developed in the terrain of the damaged lungs with *Aspergillus niger*, demonstrated by cultivation, which was treated with amphotericin (Abelcet) and voriconazole (Vfend). In November 2016, the patient was moved from another facility to the University Hospital in Hradec Kralove (Czech Republic) for further therapy. Because signs of respiratory infection were present on admission, samples from the lower respiratory tract (tracheal aspirate and BAL) were obtained, and empirical therapy with ceftazidim, levofloxacin and voriconazole was initiated. The samples from the respiratory tract microscopically demonstrated the presence of acid-fast bacilli (AFB), and the presence of *M. a.* was identified using the direct PCR method. Subsequently, *M. a.* was also confirmed in the sample by cultivation, and the isolate was further typed as *M. a*. *abscessus* (Str 2). The empirical therapy at the beginning was thus changed for a combination of amikacin, tigecycline, and clarithromycin for 3 months. Cefoxitin, which is not commonly available in the Czech Republic, was added later (selection, along with a dosage of antibiotics, according to Griffith et al., 2007 [3]). Amikacin was administered intravenously at first; however, due to a progression of amblyacusia detected during an audiological examination, inhalatory administration was applied instead. The clinical condition during hospitalization was further complicated by *Clostridioides difficile* infection (CDI), nosocomial *Pseudomonas aeruginosa* pneumonia, and continuing *Aspergillus niger* infection. After release in 2017, oral therapy with clarithromycin and clofazimine was continued. Sputum, initially cultivation positive for *M. a.*, turned negative 2 weeks after the commencement of targeted therapy, and remained such for the subsequent controls. The patient died 3 months after their release from the hospital due to the worsening GvHD.

#### 2.1.3. Case 3

A 68-year-old man was regularly monitored on an out-patient basis due to interstitial pulmonary fibrosis since the year 2015. In January 2018, during a screening examination, *M. a.* was identified by cultivation in his sputum. The isolate was further typed as *M. a*. *massiliense* (Str 3). The patient was clinically stable, without signs of infectious complications. X-ray of the lungs did not show any signs of a new pulmonary affection. The criteria of ATS [11] were not fulfilled, and a therapy of mycobacteriosis was not therefore started. Since then, *M. a.* has been repeatedly (twice a year) isolated in the samples of sputum. The patient is still clinically stabilized without a need for targeted therapy.

#### 2.1.4. Case 4

A 15-year-old boy was monitored for CF since infancy. In 2021, *M. a.* was isolated from sputum. The isolate was further determined as *M. a*. *massiliense* (Str 4). His clinical condition had been long-term stabilized and satisfactory. After a finding of *M. a.*, CT of the lungs was performed, which showed lesions supporting the diagnosis of lung mycobacteriosis (bronchiolitis and bronchiectasis). Intravenous therapy was commenced with a combination of amikacin, tigecycline, imipenem, and azithromycin for a period of 4 weeks, followed by oral therapy with azithromycin and doxycycline, and with inhalatory amikacin (selection and dosage of antibiotics according to Floto et al., 2016 [14]). Conversion of the sputum to cultivation negativity was observed 4 weeks after the initiation of targeted therapy and still continues.

#### 2.1.5. Case 5

A 24-year-old man with CF has been periodically monitored as an outpatient. In May 2021, *M. a.* was isolated from the sputum. The isolate was further identified to be *M. a*. *abscessus* (Str 5). This coincided with a worsening of dyspnea during the exertion reported by the patient. The HRCT examination of the lungs revealed a stationary finding of bronchiectases. On repeated detection of the *M. a*. *abscessus*, eradication therapy was initiated with a combination of azithromycin, imipenem, amikacin, and tigecycline for a period of 4 weeks, continuing with azithromycin, moxifloxacin, and inhaled amikacin (selection and dosage of antibiotics according to Floto et al., 2016 [14]). Conversion of the sputum to cultivation negativity appeared after 4 weeks of treatment and is continuing to this date.

### 2.2. Sequence Analysis of rpoB and rrl Genes

Sequencing examination of the *rpo*B gene of the five isolates demonstrated two isolated strains of *M. a. abscessus*, two isolated strains of *M. a. massiliense*, and one isolated strain of *M. a. bolletii*.

**Figure 1:**
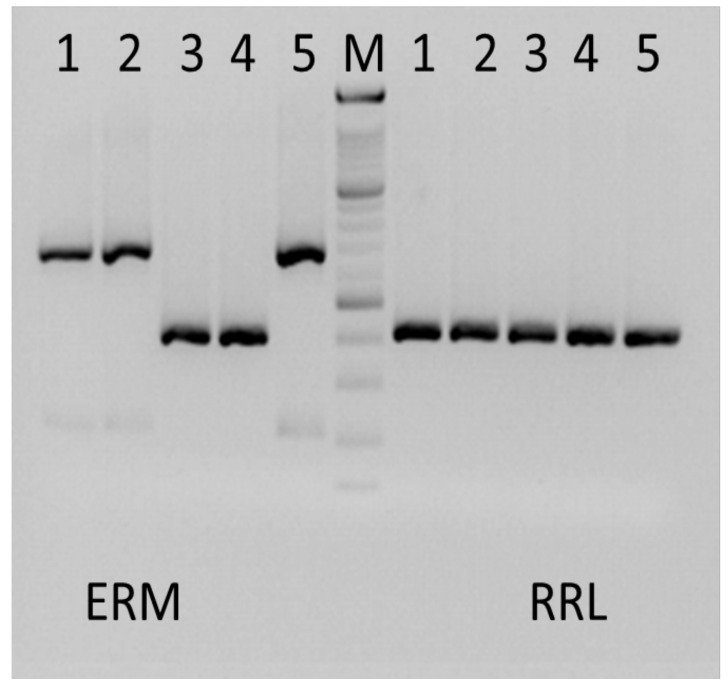
Analysis of the molecular size of the *rrl* and *erm*(41) gene products. The *rrl* and *erm*(41) gene products from isolated bacterial strains were PCR amplified for sequencing, and the size of the product was analysed in the gel. Products of the *rrl* gene (right) show in all isolates (1–5) a unified size of 420 bp. Products of the *erm*(41) gene (left) show full-length size of 673 bp in Str 1, Str 2, and Str 5, while truncated products of 397 bp were detected in Str 3 and Str 4. Molecular weight marker (M) is shown in the centre (100 bp ladder). Positive and negative controls are not shown.

The subsequent analysis of the *rrl* gene yielded PCR products of the size of 420 bp in all isolates tested (Figure 1).

Sequencing analysis of this PCR product did not show any mutations in positions 2058 and 2059, which would indicate the development of the acquired resistance to macrolides in any of the isolates (data not shown).

### 2.3. Sequence Analysis of erm(41) Gene

The full length *erm*(41) gene was demonstrated in three strains (Str 1, Str 2, and Str 5) as a resultant PCR product of the size of 673 bp (Figure 1). The presence of the complete *erm*(41) gene is typical for *M. a. abscessus* and *M. a. bolletii* [17]. In two stains (Str 3 and Str 4), a PCR product of the size of 397 bp was demonstrated (Figure 1). The presence of an incomplete *erm*(41) gene is typical for *M. a*. *massiliense*.

**Figure 2:**
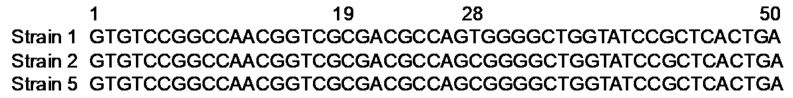
Sequence analysis of the *erm*(41) gene. Sequencing analysis of full-length *erm*(41) gene products from bacterial strains (Str 1, Str 2, and Str 5) shows “wt” T28 sequence in the Str 1 and T28→C substitution in strains Str 2 and Str 5.

Sequencing of the *erm*(41) gene was subsequently performed in all strains to verify the identification to the subspecies level. Sequencing analysis of the strains Str 1, Str 2, and Str 5 was additionally used to identify known mutations, which are responsible for the loss of the functional *erm*(41) gene, leading to the maintenance of the sensitivity to macrolides. Such specific mutation has been described for position 28, where a substitution of T for C (T28→C) [21] can occur, resulting in an amino acid substitution in codon 10 (W10R). Accordingly, isolates without this mutation, i.e., with a functional *erm*(41) gene and development of inducible resistance, are designated as T28 sequevar. Isolates with this mutation, i.e., with the non-functional *erm*(41) gene and preserved sensitivity to macrolides, are described as C28 sequevar. Another such nucleotide change has been described in position 19 (C19→T) [12], which introduces a premature stop codon instead of arginine in codon 7. The data show that Str 1 belongs to the “wt” T28 sequevar, while the strains Str 2 and Str 5 were characterized by the T28→C mutation, and thus characterized as C28 sequevar (Figure 2).

The C19→T mutation was observed in none of the isolates. These data suggest that only strain Str 1 should exhibit an inducible resistance to macrolides. The alignment of the sequences of all isolates is available in the Figure S1 (Supplementary Materials).

### 2.4. Examination of Susceptibility to Antibiotics

To confirm the sequence data and generate the overall antibiotic profile of the studied isolates, the examination of susceptibility to selected antibiotics was performed. The in vitro data showed 100% sensitivity of strains to amikacin, cefoxitin, and tigecycline. Strains also exhibited good sensitivity to imipenem, except for the Str 3. On the other hand, all the isolates were resistant to ciprofloxacin, as well as linezolid and moxifloxacin, with the exception of Str 4 and Str 5, respectively. All isolates were susceptible to clarithromycin after 3 days. However, inducible resistance developed in Str 1 during incubation for 14 days (Table 1).

## 3. Discussion

Infections with atypical mycobacteria represent an increasing problem worldwide. This study described five clinical cases of infections with *M. a*, which are otherwise relatively rare in the Czech Republic and in Europe in general.

In all five described cases, *M. a.* was isolated from the samples from the respiratory tract. The presence of NTM in the respiratory tract does not always indicate pathology, as they can colonize the respiratory tract temporarily or permanently without inducing infection [22,23]. Establishing the etiological link of these bacteria to the disease can thus be complicated, and making decisions about the necessity of the targeted therapy can be very difficult [24,25]. In general, an isolated presence of NTM in one sputum sample is not considered significant and, in such case, it is recommended to repeat the examination [11]. The detection of rapidly growing NTM from respiratory samples often represents rather transient colonization or contamination of the sample without clinical relevance [26]. Thus, the correct establishment of the diagnosis of mycobacteriosis requires a combination of laboratory, radiological, as well as clinical criteria [3].

Nevertheless, *M. a.* is a recognized causative agent of pulmonary mycobacteriosis, and its isolation from samples obtained from the respiratory tract is certainly a reason to increase attention and to monitor the clinical condition of the patient further [27,28]. Among the five patients described in this study, repeated positive cultivation findings of *M. a.* were identified as a colonization without a clinical relevance only in one patient—Case 3 (Str 3). In the other four cases, the criteria for the diagnosis of mycobacteriosis were fulfilled (Table 1), and therapy was commenced. Identification and susceptibility testing were performed in all isolates. Even patients in temporarily clinically good conditions may experience worsening of symptoms of underlying pulmonary disease, which would then require an appropriate therapy.

Described cases represent patients with another significant pathology of the lungs. Three of these cases were patients with CF, which is considered as the most significant risk factor for the development of pulmonary mycobacteriosis in children and young adults in developed countries [10]. In fact, there has been a marked increase in the cases of mycobacterioses in this population in the recent decades, with up to 20% of affected persons being positive [22]. Thus, it is important, to employ a regular screening plan for such patients—e.g., on annual basis and always during the worsening of symptoms or employing an immunomodulatory therapy. The fourth case described here was a female patient after bone marrow transplantation, which led to GvHD with a serious lung symptomatology. The last, Case 5, was a patient suffering from pulmonary fibrosis, which can also predispose for pulmonary mycobacteriosis. However, in Case 5, the criteria of ATS were not met, and the repeated detections of mycobacteria were evaluated as colonization.

The isolates in each case were further identified by DNA sequencing as *M. a*. *abscessus* and *M. a*. *massiliense* (two isolates each), and one isolate of *M. a. bolletii*. Although the number of patients in this study is small, the prevalence of the individual subspecies corresponds with published data, where *M. a.* *abscessus* is most frequently demonstrated (45–65%), followed by *M. a.* *massiliense* (20–55%), and with *M. a. bolletii* demonstrated only rarely, in 1–18% [29].

*M. a.* strains obtained in this study were examined in vitro for their susceptibility to the panel of antibiotics suitable for the treatment of mycobacterioses (Table 1). The results showed that all strains were sensitive to amikacin, cefoxitin, tigecycline, and, with an exception of one, to imipenem. On the contrary, linezolid, doxycycline, and moxifloxacin turned out to be unreliable for a potential empirical therapy, where in vitro susceptibility was observed only in two (doxycycline) and one (linezolid and moxifloxacin) of the five strains tested. Similar results were previously shown by Lyu et al. [20]. In their work, 22 isolates of *M. a. abscessus* and 16 isolates of *M. a*. *massiliense* were tested. All of these were susceptible to amikacin: 95.5% and 81.3% to cefoxitin, respectively, and 95.5% and 100% to clarithromycin after three-day incubation, respectively. We have analysed the sensitivity to clarithromycin after both 3 and 14 days to identify isolates with the inducible-resistant phenotype. Indeed, while all our strains were initially susceptible to clarithromycin after 3 days, subsequent incubation revealed the development of resistance in the strain Str 1 (*M. a. bolletii*).

Although these results indicated the resistance to be inducible, we have nevertheless performed an analysis of the *rrl* gene, which is associated with the constitutive resistance genotypic changes. As expected, none of our isolates showed mutation in this gene. These mutations are relatively rare, e.g., in the paper by Mase et al., it was detected in only one isolate out of 14 [17]. Moreover, Bastien et al. [16] described only six *rrl* mutants between 87 strains of *M. a*. These were more often isolated from cystic fibrosis patients; however, this finding was, surprisingly, not significantly associated with a previous treatment. In fact, the acquisition of *rrl* gene mutations during therapy with macrolides has been reported for several NTM species, in particular for *M. a.* [20].

The *erm*(41) gene is coding for the methylase that is capable of rendering the carrying bacteria inducible resistant to macrolides [16,17]. Thus, the sensitive/resistant phenotype of our isolates was further confirmed by the sequence analysis of this gene. Sensitivity to clarithromycin in the *M. a.* *massiliense* isolates corresponded to the fact that this subspecies carried only a truncated and thus non-functional *erm*(41) gene. While, in general, *M. a.* *abscessus* carries a functional *erm*(41) gene, some isolates remain sensitive to macrolides as a result of a loss of the functional *erm*(41) gene due to point mutations, the frequency of which is described at about 15–20%. Two kinds of such loss-of-function mutations have been described so far. Thus, T28C mutation leads to the substitution of tryptophan for arginine in codon 10, and such strains are called C28 sequevars. Another mutation C19T introduces a premature stop codon (T19 sequevar) [12]. Our analysis showed that both our *M. a*. *abscessus* isolates were of the C28 sequevar and, thus, are sensitive to macrolides. Only the *M. a. bolletii* (Str 1) in our study contained the functional *erm*(41) gene (sequevar T28), which resulted in its resistance to clarithromycin in vitro after 14 days. Accordingly, the antibiotic therapy of this patient was the most complicated and lasted for 4 years.

In general, mycobacterioses represent serious diseases, which are inherently difficult to treat [30]. Currently, there are no reports of controlled clinical trials of treatment for *M. a*. mycobacterioses. Treatment recommendations are based on case series, in vitro susceptibility testing, and the clinical experience of experts [14]. Combinations of antibiotics comprising macrolides as a cornerstone are recommended for the treatment of *M. a.* infections. These can, however, frequently fail due to the development of resistance to macrolides by the mechanisms described above [27]. The usually better therapeutic results are thus achieved in the treatment of infections caused by the subspecies *M. a*. *massiliense*, carrying the non-functional *erm*(41) gene [28,31,32]. This is further illustrated by larger studies. Thus, Jeon et al. [27] described treatment efficacy in a large cohort of 65 patients suffering from pulmonary mycobacterioses caused by *M. a*. The patients were initially treated for a period of one month with intravenous amikacin and cefoxitin and oral clarithromycin. In the continuation stage, the combination of clarithromycin and doxycycline was used with the median duration of therapy being 24 months. As a result, the clinical recovery occurred in 83% patients, and conversion of the sputum to cultivation negativity occurred in 58%. However, among these, the conversion was significantly lower in patients infected with strains with demonstrated resistance to macrolides (17% versus 64%). On the other hand, when a group of patients was treated for subsp. *massiliense* infection, conversion of the sputum to cultivation negativity already occurred in the course of the first month of therapy, indicating the importance of the non-functional *erm*(41) gene in this subspecies [20]. This was further confirmed by the improved efficacy of the eradication of *M. a*. *abscessus* in those patients, who were infected by the sequevar C28 carrying the non-functional *erm*(41) gene [12]. Similarly, Choi et al. [33], in their paper, describe 14 patients with pulmonary affection induced by *M. a.* *abscessus*, where all 14 isolates possessed a non-functional *erm*(41) gene, and the conversion of sputum to cultivation negativity was achieved in 93% of patients.

In three of our four treated patients, cultivation negativity was observed after the first 4 weeks of therapy. The repeated cultivation positivity was observed only in a patient with *M. a. bolletii* infection (Case 1), which was the only strain resistant to macrolides in our cohort. Case 1 was successfully managed by an extended therapy lasting 4 years, which was partly also due to a poor compliance of the patient. Patient compliance during the long-term antibiotic therapy is essential, as any misuse of antibiotics (failure to adhere to dosing intervals, complete omission of therapy) can lead to the therapy failure. In this case, conversion of the sputum to cultivation negativity occurred after an improvement of patient’s compliance and the addition of clofazimine to the therapeutic regime.

In the Case 2 patient, the result of therapy could not be evaluated, as she died after several months due to the GvHD refractory to therapy. The infections in the remaining two patients (Cases 4 and 5) were caused by strains susceptible to macrolides, and the introductory therapy led to both the clinical improvement and the conversion of the sputum to cultivation negativity after 4 weeks. These cases are, however, recent, and the continuation therapy is still ongoing (Case 4: entire therapy for 10 months, and Case 5: entire therapy for 8 months).

*M. a.* infections represent emerging infections in patients with CF or other chronic respiratory diseases [16]. At present, *M. a.* is usually identified only at the species level in routine laboratory practice [9,34]. In our laboratory, we introduced PCR methods for the subspecies level identification. However, the results showed that identification to the level of subspecies could still be insufficient, and that more detailed molecular genetic analysis is necessary for epidemiological studies of circulating strains [35,36]. Recently, molecular analysis of the *erm*(41) gene and its sequencing was described as a powerful tool for the prediction of macrolide susceptibility and the quick determination of the initial therapeutic strategy [13]. Results of the analysis of the *erm*(41) gene correlate well with MIC values [37], which was also confirmed in our study. All five *M. a.* strains were isolated from patients living in the East Bohemian region between years 2013 and 2021. In this study, the *erm*(41) gene analysis revealed a unique representation of *M. a.* strains, since, in two of them, an incomplete gene associated with macrolide susceptibility was found. Other two strains with complete but non-functional *erm*(41) genes were assigned as C28 sequevars. In only one strain, the inducible resistance was also proved (T28 sequevar). The correlation between *erm*(41) analysis and susceptibility determination to clarithromycin was excellent. Moreover, the *erm*(41) analysis significantly shortens the decision time for macrolide therapy, because inducible resistance is not detected until after 14 days. Although our group of patients is small, it represents all cases of *M. a.* infections identified in the Czech Republict to this date. Thus, it provides first record of this infection in the country and describes an obvious association between results of *erm*(41) gene analysis and clinical outcome.

## 4. Materials and Methods

Clinical samples (sputum, tracheal aspirate, and bronchoalveolar fluid) were cultivated on solid media for 6 weeks at 37 °C under aerobic condition (Löwenstein–Jensen and Ogawa media; Labmediaservis, Jaromer, Czech Republic) and for 6 weeks at 37 °C in MGIT test tubes with modified Middlebrook broth in a semiautomatic system Bactec MGIT 960 (Becton Dickinson, Sparks, MD, USA).

Identification of *Mycobacterium* species was performed by molecular typing. Nucleic acids were isolated directly from the suspension of suspect isolates or respiratory tract samples using a QIAamp DNA Mini Kit (Qiagen, Hilden, Germany) according to the modified protocol for mycobacteria provided by the manufacturer.

DNA of suspected isolates was identified as the species *Mycobacterium* using the PCR method (Anyplex MTB/NTM Assay, Seegene, Seoul, Republic of Korea). In case of positivity, further identification of *Mycobacterium* involved PCR amplification and sequencing by capillary electrophoresis of *rpo*B gene (ABI 3500, Genetic Analyzer, Applied Biosystems/Thermo Fisher Scientific, Foster City, CA, USA) as previously described [38]. Final sequences were subsequently analysed with Bionumerics 7.6.2 (Applied Maths, Ghent, East Flanders, Belgium). The nucleotide sequence analysis showed the best BLAST hit with *M. a.* The *M. a.* subspecies were further typed by *erm*(41) gene amplification and sequencing according to Mase et al., 2011 [17]. The amplification of the *erm*(41) gene with an incomplete product (product size 397 bp) identified *M. a. massiliense*. The full length *erm*(41) gene products (size 673 bp) indicated *M. a. abscessus* or *M. a. bolletii*, and further typing was based on the variability of nucleotides in select positions, as previously reported and shown in Table 2 [16,17].

Subspecies identification of *M. a.* was further confirmed by PCR method based on variable-number, tandem-repeat analysis, as previously described by Choi et al., 2011 [39]. Amplification and sequencing analyses of *erm*(41) and *rrl* genes (according to Inagaki et al. [40]) were used for resistance determination.

PCR assays for *erm*(41) and *rrl* genes were performed in a volume of 25 µL containing buffer TaKaRa, 200 nM of dNTPs, 500 nM primers, and 1.0 U HS Taq polymerase TaKaRa. The amplification parameters were as follows: for *erm*(41) gene: 95 °C/10 min; followed by 95 °C/30 s, 60 °C/30 s, and 72 °C/30 s (35 cycles) finished by 72 °C/7 min and for *rrl* gene: 95 °C/10 min; followed by 94 °C/60 s, 55 °C/60 s, and 72 °C/60 s (35 cycles) finished by 72 °C/7 min. PCR products of both genes were visualized in agarose gel using an ethidium bromide. As positive control, the reference EQA sample of *M. a.* was used. Method was also validated on External Quality Assessment (EQA)—Quality Control for Molecular Diagnostics (QCMD) samples, where *M. a.* was correctly identified. Amplified DNA fragments of *erm*(41) and *rrl* genes were directly sequenced by capillary electrophoresis using primers identical to those used for PCR (ABI 3500, Genetic Analyser Applied Biosystems/Thermo Fisher Scientific, Foster City, CA, USA). The obtained nucleotide sequences were compared to reference sequences from GenBank (for *M. a. abscessus* FJ358485, for *M. a. bolletii* FJ358491 and for *M. a. massiliense* FJ358487) in an online tool, available at www.ebi.ac.uk/Tools/psa/emboss_needle. Sequence alignments were performed using DNAstar Lasergene software v. 17.3 (DNAstar, Madison, WI, USA), utilizing the Clustal W algorithm.

Susceptibility of the isolates to antibiotics was examined by means of the E-test and the broth microdilution method. The results were interpreted according to the Clinical and Laboratory Standards Institute (CLSI). Selection of antibiotics included amikacin, cefoxitin, imipenem, clarithromycin, linezolid, doxycycline, tigecycline, ciprofloxacin, and moxifloxacin.

MIC values of amikacin, cefoxitin, linezolid, doxycycline, ciprofloxacin, and moxifloxacin were established using the broth microdilution method with 96-well microtiter plates. Cation-adjusted Mueller–Hinton media (Becton Dickinson, Sparks, MD, USA) was used. Fresh colonies were harvested from the surface of solid media to saline. The bacterial suspension was homogenized on a vortex and diluted to a 0.5 McFarland standard density. The inoculum was prepared by a 1:100 dilution of 0.5 McFarland suspension in cation-adjusted Mueller–Hinton broth and added to plates.

MIC values of imipenem, clarithromycin, and tigecycline were performed by E-test (bioMérieux, Marcy l’Etoile, France) on Mueller–Hinton agar plates (Thermo Fisher Scientific, Basingstoke, UK). The bacterial suspension was prepared as above, and inocula were applied by swabbing according to Biehle et al. [41]. The plates were read after 3 days of incubation period. The MIC of clarithromycin was read again after 14 days according to Koh et al. [18].

## 5. Conclusions

Diseases caused by *M. a.* rank among the most difficult mycobacterioses to treat. The recommended treatment consists of a combination of antibiotics; however, the therapy can often fail due to the development of resistance to the key antibiotic, macrolide. The correct indication to the level of subspecies, testing of suitable antibiotics, and evaluation of sensitivity to clarithromycin after a prolonged incubation period are of principal importance for their management. Molecular detection of the *erm*(41) gene products and its sequencing can reliably and rapidly demonstrate the possibility of development of inducible resistance and thus influence the extent of introductory therapy and its duration. Our results show that the identification of the *M. a.* isolate to the level of subspecies is not necessarily sufficient. Only further analysis of the *erm*(41) gene in both isolates of *M. a*. *abscessus* allowed for the detection of the mutation, which resulted in the preserved sensitivity to macrolides. While this study shows only a limited number of cases, two out of five isolates (40%) carry a non-functional gene for the development of inducible macrolide resistance. This indicates that the share of such isolates with the preserved sensitivity to macrolides may be larger in such patients in our population than has been published so far. This should be considered when designing treatment strategies for the *M. a.* infections.

## Figures and Tables

**Table 1:** Susceptibility to antibiotics and clinical relevance of *Mycobacterium abscessus* strains.

Diagnostics/Antibiotics/	*M. a. bolletii*	*M. a. abscessus*		*M. a. massiliense*	
Therapeutics Outcome	Case 1 (Str1)	Case 2 (Str2)	Case 5 (Str5)	Case 3 (Str3)	Case 4 (Str4)
Diagnostics	CF	GvHD, RI	CF	IPF	CF
Clinical relevance ^1^	Yes	Yes	Yes	No	Yes
Clarithromycin	1 (3 d) 64 (14 d)	0.125 (3 d) 1 (14 d)	0.125 (3 d) 0.125 (14 d)	0.125 (3 d) 0.125 (14 d)	1 (3 d) 1 (14 d)
Amikacin	8	8	2	8	1
Imipenem	4	8	1	32	2
Linezolid	>256	>256	16	24	8
Tigecycline	0.25-2	0.38	0.25	0.25	0.5
Ciprofloxacin	8 > 32	>32	2	16	2
Moxifloxacin	>32	>32	1	8	2
Cefoxitin	8	8	8	16	16
Doxycycline	4	>8	128	>8	4
Time to conversion of sputum to negativity	4 years	2 weeks	4 weeks	Not	4 weeks
Therapeutics outcome	Recovered	Died	Treated	Not treated	Treated

MIC = Minimum Inhibitory Concentration; *M. a.* = *Mycobacterium abscessus*; Str 1 = Strain 1; CF = cystic fibrosis; GvHD = Graft versus Host Disease, RI = respiratory infection; IPF = interstitial pulmonary fibrosis; ^1^ clinically relevant repeated isolation of *M. a.* was evaluated; in Case 3, the repeated isolation was not clinically relevant, and treatment was not required. d = day.

**Table 2:** Sequence differences identifying *M. a.* subspecies.

*erm*(41) Gene	*M. a. abscessus*	*M. a. bolletii*	*M. a. massiliense*
Amplicon size	673 bp	673 bp	397 bp (deletion at nucleotides 64, 65 and 159–432)
Promoter sequence at position −35	T**A**TCGA	T**G**TCGA	T**G**TCGA
Nucleotide at position 28	T or C	T	T
Nucleotide at position 312	A	C	-
Nucleotide at position 336	T	C	-

## Data Availability

Data are available on request due to the ethical restrictions.

## Supplementary Materials

The following supporting information can be downloaded at: https://www.mdpi.com/article/10.3390/antibiotics11070873/s1, Figure S1: Sequence alignments of the individual *M. abscessus* strains.
