# Supplementary figures and images for: Mycobacterioses Induced by Mycobacterium abscessus: Case Studies Indicating the Importance of Molecular Analysis for the Identification of Antibiotic Resistance
Source: Antibiotics (Basel). 2022 Jun 28;11(7):873. doi: 10.3390/antibiotics11070873 (PMC9312086; doi:10.3390/antibiotics11070873)

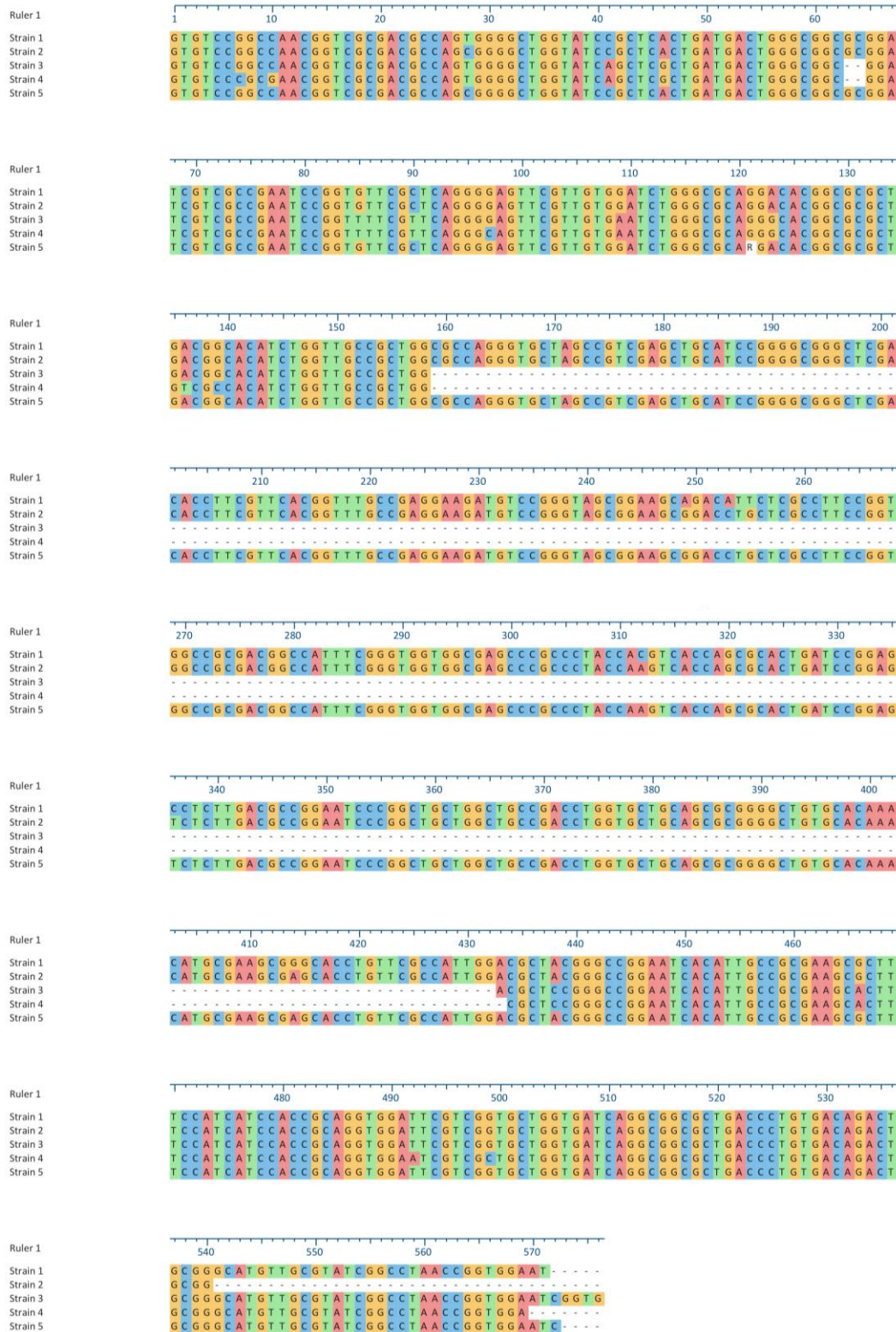

**Figure S1.** Sequence alignments of the individual *M. abscessus* strains.

Supplement: Supplementary file 1 [file antibiotics-11-00873-s001.zip › antibiotics-1761044-supplementary.pdf]
